# Factors Influencing Duration of Breastfeeding: Insights from a Prospective Study of Maternal Health Literacy and Obstetric Practices

**DOI:** 10.3390/nu16050690

**Published:** 2024-02-28

**Authors:** Rafael Vila-Candel, Francisco Javier Soriano-Vidal, Cristina Franco-Antonio, Oscar Garcia-Algar, Vicente Andreu-Fernandez, Desirée Mena-Tudela

**Affiliations:** 1 Faculty of Health Sciences, Universidad Internecinal de Valencia (VIU), 46002 Valencia, Spain; rafael.vila@professor.universidadviu.com; 2 La Ribera Primary Health Department, 46600 Alzira, Spain; 3 Foundation for the Promotion of Health and Biomedical Research in the Valencian Region (FISABIO), 46020 Valencia, Spain; 4 Department of Obstetrics and Gynecology, Xàtiva-Oninyent Health Department, 46800 Xàtiva, Spain; 5 Department of Nursing, Universitat de València, 46007 Valencia, Spain; 6 Department of Nursing, Universidad de Extremadura, 10003 Cáceres, Spain; 7 Neonatology Unit, ICGON, Hospital Clinic-Maternitat, BCNatal, 08028 Barcelona, Spain; ogarciaa@clinic.cat; 8 Instituto de Investigaciones Biosanitarias, Universidad Internacional de Valencia (VIU), 46002 Valencia, Spain; vicente.andreu@professor.universidadviu.com; 9 Department of Nursing, Instituto Universitario de Estudios Feministas y de Género Purificación Escribano, Universitat Jaume I, 12071 Castellón de la Plana, Spain; dmena@uji.es

**Keywords:** breastfeeding, health literacy, abandonment, nursing

## Abstract

Numerous factors concerning early breastfeeding abandonment have been described, including health literacy (HL). This study’s objective was to analyze factors related to early breastfeeding abandonment (<6 months). This prospective multicentric study examined the duration of breastfeeding at 6 months postpartum and was conducted in four different regions of Spain from January 2021 to January 2023. A total of 275 women participated in this study, which focused on maternal HL and obstetric practices. A decrease in the breastfeeding rate was observed from hospital discharge (*n* = 224, 81.5%) to the sixth month postpartum (*n* = 117, 42.5%). A Cox regression analysis revealed that inadequate HL levels, lack of mobilization during labour, and induced labour were significantly associated with early breastfeeding cessation (*p* = 0.022, *p* = 0.019, and *p* = 0.010, respectively). The results highlight that women with adequate HL had a 32% lower risk of early breastfeeding abandonment. In comparison, mobilization during labour and induction of labour were linked to a 32.4% reduction and a 53.8% increase in this risk, respectively. These findings emphasize the importance of considering obstetric and HL factors when addressing the breastfeeding duration, indicating opportunities for educational and perinatal care interventions.

## 1. Introduction

Breastfeeding (BF) is a health-promoting behaviour [1]. Furthermore, the associated relationship between mother and baby goes beyond mere nourishment [2]. Despite its notable and numerous physical, emotional, and psychological benefits and the significant role that BF plays in maternal and infant health in the short, medium, and long term, BF rates remain improvable.

International organizations, such as the World Health Organization (WHO) and the United Nations International Children’s Emergency Fund (UNICEF), recommend maintaining exclusive breastfeeding (EBF) for at least the first six months of infants’ lives. However, according to data, it is estimated that globally, 43.8% of infants under 6 months are exclusively breastfed [3]. In Europe, this figure rises to 60%, but it is unknown whether it pertains to EBF or any other form of breastfeeding [4]. It is also important to note significant variability in the data depending on the European country of origin. In Spain, the national health survey in 2017 showed that the percentage of EBF at 6 months was 39% [5]. However, other studies have reported significant variability between cities and autonomous communities. For example, the reported EBF rate in Madrid was 25.4% [6], 16.8% in Catalonia [7], and 21.6% in the Vasque Country [8]. The latest multicentre study that reported figures in Spain indicates that 57.3% of women maintained breastfeeding, including EBF and mixed feeding, up to 6 months postpartum [9]. In order to contribute to improving these figures, it is relevant to understand the factors that influence the duration of breastfeeding to promote optimal practices.

Health literacy (HL) has been defined as “The ability of an individual to obtain and translate knowledge and information in order to maintain and improve health in a way that is appropriate to the individual and system contexts” [10]. Furthermore, HL is understood to be a continuous learning process that requires the ability to access, comprehend, critically evaluate, and apply health-related information [10,11]. Among the many published articles, in 2001, Kaufman [12] was the first to establish a correlation between HL and the maintenance of BF. Subsequent studies, however, have presented diverse outcomes, with the anticipated link between HL and BF maintenance not uniformly affirmed in all instances. This variability may stem from the specific characteristics of the study population, or the nuances of the screening tools employed, which are often adapted from languages other than the one under investigation. Consequently, this underscores the imperative for meticulously scrutinizing contextual and methodological elements in deciphering the association between HL and BF duration.

The limited comparability with other studies emanates from incongruent definitions of BF outcomes and the myriad methods utilized to assess HL. Researchers [12,13,14] employed questionnaires, such as the Short Test of Functional Health Literacy in Adults or Rapid Estimates of Adult Literacy in Medicine, to measure HL. Despite the divergent HL assessment approaches, a consistent finding emerged, which revealed positive correlations between HL and BF behaviour. Nevertheless, the distinct criteria used to assess BF outcomes introduce additional intricacies into direct comparisons. Conversely, other authors [15,16] have reported no statistically significant association between functional HL, as evaluated using the Newest Vital Sign screening tool, and EBF for more than 4 months. Considering the observed variability in results, our study is positioned as a valuable addition to the existing body of evidence. However, recognizing that maternal HL levels may influence the understanding and adherence to BF recommendations, further research is warranted.

Historically, it has been observed that the medicalization of childbirth significantly impacted BF rates. At the beginning of the 20th century, most births occurred at home, and a breastfeeding culture was well-established, with knowledge transmitted effectively between women; this context resulted in high breastfeeding rates [17]. In contrast to this, the model of care centred on medical authority led to barriers in breastfeeding related to obstetric practices, such as the separation of the mother–child dyad during the clinical postpartum period [17,18]. Currently, we know that mother–baby separation after birth, the excessive use of medical interventions during childbirth, or a lack of support during the clinical postpartum period are practices that do not favour the establishment and maintenance of breastfeeding. On the other hand, despite high levels of intervention, as in a surgical procedure, like a Caesarean section, it is known that respecting dyad practices, such as early skin-to-skin contact or early and spontaneous breastfeeding initiation, favour the establishment and long-term maintenance of breastfeeding [19,20,21].

As breastfeeding practices significantly contribute to infant health and development, unravelling the intricate relationship between maternal HL, obstetric practices, and BF duration holds the potential to guide evidence-based approaches for promoting and sustaining optimal breastfeeding practices. Therefore, this study aimed to analyze factors related to early breastfeeding discontinuation (<6 months).

## 2. Materials and Methods

### 2.1. Study Design

This multicentre prospective study was conducted in four public hospitals across Spain from January 2021 to January 2023.

### 2.2. Participants and Study Area

Women meeting the eligibility criteria were enrolled in primary health centres between 24 and 37 weeks of pregnancy. This study strategically chose four hospitals that were geographically dispersed—three in the east and one in the west of Spain—to ensure a diverse analysis. This inclusive approach facilitated result generalization while mitigating biases. Specifically, the eastern region included the General Hospital of Castellón (northeast (H3)), Hospital de la Ribera (H1), and Hospital Lluis Alcanyis (southeast (H2)), with comparable annual deliveries. The General Hospital of Cáceres (H4) in the west of Spain offers maternity care to women with distinct characteristics. All four hospitals share similarities in birth rates, treated prematurity, and participation in the IHAN program for maternity healthcare quality. Collectively serving 500,000 people, they witness approximately 5000 births annually, with pregnant women recruited during their third trimester from primary care clinics managed by affiliated midwives.

### 2.3. Inclusion and Exclusion Criteria

Participants were enrolled during the third trimester of pregnancy in the midwifery-led primary care consultations in each participating centre. The inclusion criteria were women who accepted and signed the informed consent form, had Internet access, and intended to breastfeed.

This study’s exclusion criteria were females under 16 years of age; individuals with cognitive impairments, language barriers, or illiteracy (unable to read in Spanish); newborns with congenital malformations; and multi-child pregnancies.

### 2.4. Sample Size

We estimated the necessary sample size based on an annual population of 5000 births across the 4 participating hospitals, assuming a 65% discontinuation rate of breastfeeding at 6 months, with a significance level of 0.05% and a power of 90%, along with an estimated 10% loss to follow-up. The total sample size calculated was 261 participants.

### 2.5. Baseline Variables

The baseline data collection encompassed the following variables:

- Sociodemographic variables: maternal age, country of origin (Spain/foreign), level of education (primary to secondary school/university), employment status (professional to employee/unemployed/student), civil status (married/others), economic status (<EUR 1000 per month/>EUR 1000 per month), and financial status (bad–regular/good–very good).
- Health-literacy-related variables: HLS-EU-Q16, which assesses the population’s HL through a Likert scale with 16 items according to “very easy (1 point)”, “easy (1 point)”, “difficult (0 points)”, and “very difficult (0 points)”. This unifactorial scale exhibits good internal consistency, with a McDonald’s omega value of 0.982 in the Spanish population [22]. Level of HL: adequate (>12 points) or inadequate (≤12 points) (Supplementary Table S1).
- Obstetric–neonatal variables: gestational age at birth, parity (nulliparous/multiparous), type of onset of labour (spontaneous or elective Caesarean section/induced), type of rupture of membranes (spontaneous/artificial), group B streptococcus status (positive/negative), intrapartum antibiotic use (yes/no), intrapartum analgesia (inhalatory/local/epidural/none), Kristeller manoeuvre (yes/no), completion of birth (spontaneous vaginal/instrumental (vacuum, spatulas, forceps)/Caesarean section), episiotomy (yes/no), perineal condition following birth (intact/grade 1/grade 2/grade 3/grade 4) [23], newborn gender (female/male), newborn weight (grams), early skin-to-skin contact [(within 30 min and lasting for at least 2 continuous hours) (yes/no/with father)], early start of breastfeeding (within 2 h/after more than 2 h), drinking allowed during labour (yes/no), accompaniment of maternal choice allowed (yes/no), mobilization allowed during labour (yes/no), and positioning at the moment of birth (vertical/lying down—lithotomy position/lateral decubitus).
- Response variable: type of nursing (BF/supplementary feeding (SF)/mixed feeding (MF)) at 6 months postpartum, assessing the newborn and infant feeding practices. The response variable “Suspension of BF at 6 months” (yes/no) considered whether the infant was receiving SF (“yes”) or continued with BF or MF (“no”) at 6 months.
- Variables related to previous breastfeeding education: information/training in breastfeeding (none/previous information received from relatives; friends; or health professionals, such as midwives, pediatric nurses, obstetricians, and paediatricians); consultation of texts; participation in birth preparation groups, nursing groups, or postpartum groups; and the use of digital tools.

### 2.6. Data Collection

A web platform was developed for study monitoring in each of the four cohorts in Spain: Hospital de la Ribera (H1), Hospital Lluis Alcanyis (H2), General Hospital of Castellón (H3), and General Hospital of Cáceres (H4), all of which had comparable annual birth rates. After recruitment and electronic acceptance of the informed consent form, the participants received a survey via email based on the expected due date. In the initial baseline survey, all sociodemographic data and health literacy levels were collected using the screening tool HLS-EU-Q16. After childbirth, each participant received surveys at 15 days, 6 weeks, 3 months, and 6 months postpartum. Collaborating researchers from each health department of the 4 regions (H1, H2, H3, and H4) were given secure access to the platform to record birth data and the number of visits made by various healthcare professionals during the study period. The collected information was entered into an electronic database while ensuring compliance with current regulations and guaranteeing confidentiality and anonymity. Losses and dropouts during this study and their causes were recorded. However, researchers were not authorized to view the planned surveys that the participants completed during the study follow-up. Finally, the data manager was responsible for matching the participants’ survey responses with their birth dates and the follow-ups performed by various healthcare professionals for up to 6 months. This approach ensured confidentiality and complied with data protection regulations. Our methodology prioritized user anonymity and data security, which allowed for accurate matching while safeguarding participants’ privacy rights.

### 2.7. Data Analysis

The dataset underwent comprehensive descriptive analyses, which involved examining the distinctive features of each variable. Statistical tests, such as Fisher’s test or *t*-test, were selectively applied to compare means. Bivariate comparisons scrutinized the early breastfeeding abandonment (<6 months) (yes/no) at multiple time points, including at discharge, 15 days, 6 weeks, 3 months, and 6 months, while considering sociodemographic, health literacy, and obstetric–neonatal variables through the chi-square test. Additionally, survival analysis using the Kaplan–Meier method gauged the statistical significance of variables related to early breastfeeding abandonment over 6 months. A Cox regression model was formulated and incorporated statistically significant variables. 

The statistical analysis was performed using SPSS v. 28.1 for Windows (IBM Corp. 2018, Armonk, NY, USA), with a significance threshold set at *p* < 0.05.

## 3. Results

Out of a total of 280 women, 5 were excluded for the following reasons: 2 perinatal deaths and 3 lost to follow-up. The total analyzed sample consisted of 275 participants. A total of 44.7% (123/275) of the births were attended at H1, 27.3% (75/275) at H4, 15.6% (43/275) at H3, and 12.4% (34/275) at H2. Table 1 presents a chi-square analysis to assess the associations between various variables and early BF abandonment. The chi-square test was applied by comparing each category’s observed and expected frequencies, with results stratified by the responses (no or yes) regarding early BF abandonment. The associated *p*-values indicate the statistical significance of these associations. Notably, the comparisons were made by analyzing the table’s columns.

The mean age of participants was 33.2 ± 4.4 years (*p* = 0.977), with 90.2% (248/275) being Spanish-born women (*p* = 0.308) (Table 1). Most participants had a university education (53.8%, *n* = 148/275; *p* = 0.994), were married (66.5%, *n* = 183/275; *p* = 0.994), were employed (64.7%, *n* = 178/275; *p* = 0.994), had an adequate level of income (54.9%, *n* = 151/275; *p* = 0.499), and perceived good or very good economic stability (51.3%, *n* = 141/275; *p* = 0.142). All women desired to breastfeed, with 90.2% (248/275) aiming for EBF, 6.5% (18/275) opting for MF, and the rest undecided (*p* = 0.177). Information on BF was primarily received from family and friends (24.7%, *n* = 68/275), healthcare professionals (25.5%, *n* = 70/275), and digital tools (25.1%, *n* = 69/275) (*p* = 0.093). Approximately, 76.4% (210/275) of pregnancies were classified as low risk, without differences between groups (*p* = 0.649). The mean gestational age at birth was 39.3 ± 1.2 weeks (*p* = 0.475), 73.4% were primiparous (202/275; *p* = 0.172), and the mean birth weight was 3254 ± 401 g (*p* = 0.494). The induction rate was 29.1% (80/275), with 37.4% (103/275) undergoing artificial rupture of membranes. Women who discontinued breastfeeding early had a higher rate of induced labour and artificial rupture of membranes (*p* = 0.014 and *p* = 0.013, respectively). Most women were negative for group B Streptococcus (81.8%, *n* = 225/275; *p* = 0.931); received epidural analgesia (78.2%, *n* = 215/275; *p* = 0.420); had a spontaneous vaginal birth (56.7%, *n* = 156/275; *p* = 0.443); and had no episiotomy (54.2%, *n* = 149/275; *p* = 0.484), with 16.7% (46/275) having an intact perineum without differences between groups (*p* = 0.846). During labour, 69.1% (190/275) were allowed to drink, their partner accompanied in 94.9% (261/275; *p* = 0.257) of cases, and 69.1% (190/275) could move during dilation, with the lithotomy position used for birth in 51.6% (142/218, excluding C-sections; *p* = 0.087). Statistically significant differences were observed regarding early BF discontinuation, with a higher percentage of women not allowed to drink (*p* = 0.031) and those with restricted mobility (*p* = 0.019). Maternal skin-to-skin contact (SSC) was performed in most cases (89.1%, *n* = 245/275; *p* = 0.288), with early initiation of breastfeeding in 72.4% (199/275; *p* = 0.146). No statistically significant differences were found between the key variables or with the predictor variables of the model presented according to the women’s hospital of origin.

The HL level showed that 69.5% (191/275) of women had an adequate level. Statistically significant differences were observed between the HL level and early breastfeeding discontinuation, with women that had inadequate levels discontinuing breastfeeding at a higher rate at all cutoff points: at discharge (*p* = 0.031), at 15 days (*p* = 0.025), at 6 weeks (*p* = 0.017), at 3 months (*p* = 0.012), and at 6 months (*p* = 0.04). No statistically significant differences were observed between the HL level and the different sociodemographic variables, such as country of origin (*p* = 0.323), educational level (*p* = 0.400), marital status (*p* = 0.255), employment status (*p* = 0.231), economic status (*p* = 0.178), and financial stability (*p* = 0.239). 

**Figure 1:**
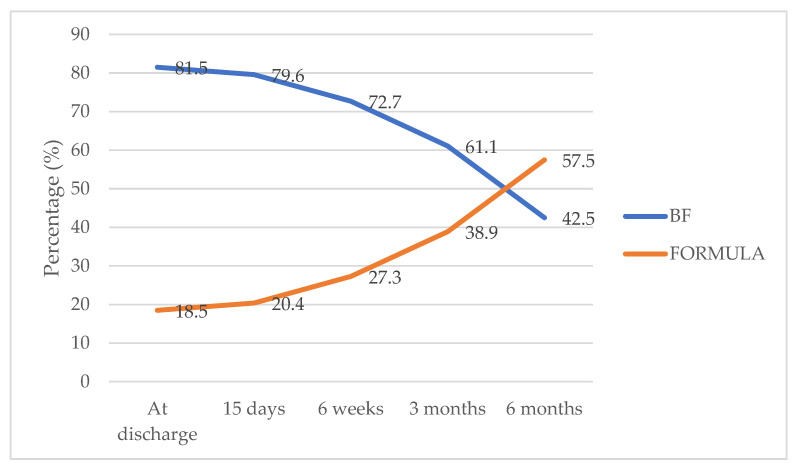
Rates of breastfeeding during the study follow-up (N = 275).

Regarding the type of breastfeeding, we can observe in Figure 1 a reduction from 81.5% (224/275) at discharge to 42.5% (117/275) at 6 months postpartum. The mean time of BF duration was 108.1 ± 72.8 days.

**Figure 2:**
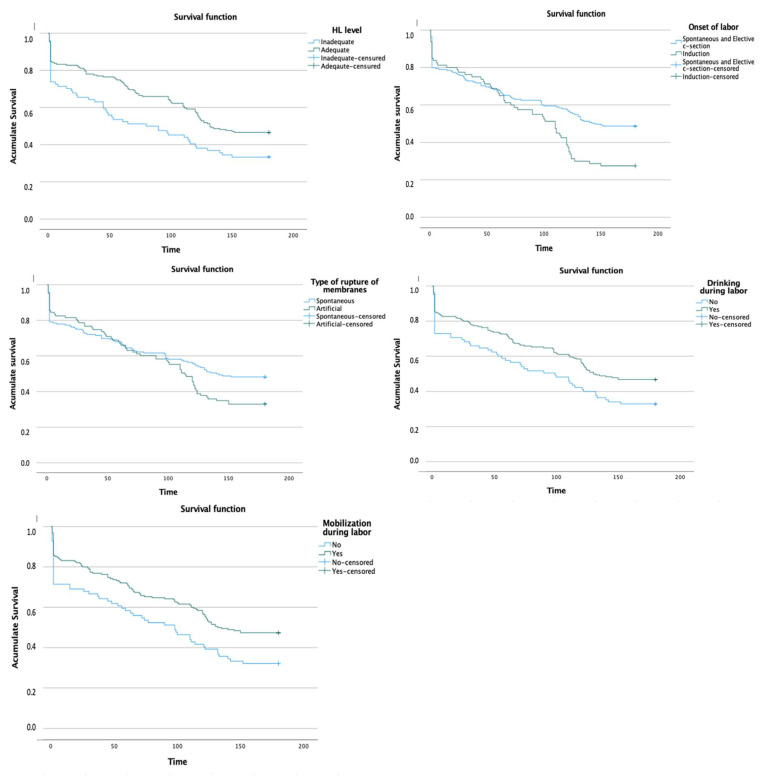
Survival curves for early breastfeeding abandonment based on statistically significant variables. Each curve corresponds to a distinct group within the variable, portraying the cumulative probability of participants within that group continuing to breastfeed over time. The *x*-axis denotes time, and the *y*-axis represents the proportion of women maintaining breastfeeding at each time point. Disparities between the curves signify variations in the likelihood of breastfeeding continuation between the compared groups.

We were interested in analyzing the correlation between the average time until early abandonment of BF and variables that showed statistical significance in the bivariate analysis (Table 2 and Figure 2). Additionally, we present the results of Kaplan–Meier survival models used to analyze the BF duration based on statistical variables in the bivariate analysis. The log-rank test (Mantel–Cox) was applied to assess differences in survival functions between the compared groups and determine the statistical significance of these differences. Significant differences were observed in the mean breastfeeding duration between health literacy levels (*p* = 0.010), type of onset of labour (*p* = 0.004), type of rupture of membranes (*p* = 0.046), fluid intake during labour (*p* = 0.019), and mobilization during labour (*p* = 0.009). 

Survival curves, which were generated using the Kaplan–Meier method, provide visual information about the probability of an event occurring over time. These curves, as shown in Figure 2, show the probability of maintaining BF without early abandonment as time progressed (as represented on the *x*-axis) from initiation. We observed differences over time, with early abandonment of BF occurring earlier in women with inadequate HL, induced labour, artificial rupture of membranes, inability to drink during labour, and lack of mobility during labour.

Finally, the Cox regression analysis assessed the association between specific variables and the BF duration. We employed a Cox regression model to investigate the multiple factors that influenced the duration of BF, with a particular focus on significant variables in the survival analysis. The results, as presented in Table 3, elucidate the predictive value of the HL level, mobilization during labour, and the type of onset of labour. 

The coefficient for the HL level was −0.384 (*p* = 0.022), indicating a statistically significant association. The Exp(B) value of 0.681 suggests that women with an adequate HL level had an approximately 32% lower risk of early BF abandonment compared with the reference group.

Mobilization during labour demonstrated significance, with a coefficient of −0.392 (*p* = 0.019). The corresponding Exp(B) value of 0.676 indicates a 32.4% reduction in the risk of early BF abandonment for women who were allowed mobilization during labour.

The type of onset of labour exhibited significance with a coefficient of 0.431 (*p* = 0.010). The Exp(B) value of 1.538 suggests a 53.8% increase in the risk of early BF abandonment for induced labour compared with the reference group.

These findings underline the importance of HL, mobilization during labour, and the type of onset of labour as significant predictors of BF duration. 

## 4. Discussion

Our study results suggest the influence of certain variables on breastfeeding practices, highlighting the importance of obstetric and socio-educational considerations in promoting BF, with less abandonment found when women had adequate HL, labour was not induced, membranes were ruptured spontaneously, and the ability to drink and mobilize was present during labour.

As in previous studies in our country [6,7,8], the rates of BF in the sixth month did not reach those recommended by international organizations, such as the WHO [24], with rates in our sample being lower than those reported by other studies conducted in our country [9]. However, compared with rates in the rest of Europe, Spain obtained similar figures for BF at six months [25]. It is essential to bear in mind that our analysis of the BF rate included both exclusive and mixed breastfeeding. Therefore, the results obtained were lower than the proposed target rates.

In our study, we observed that various factors had a negative impact on the continuation of BF. HL is one of the most significant factors in determining whether BF is continued or abandoned early. This association has been observed by different authors using various screening tools, leading to heterogeneous results [13,16,26]. In our case, we used a validated tool adapted to Spanish with an alpha coefficient of 0.982 [22]. 

Various variables influencing maternal HL have been described, such as educational level and economic status [27]. No socioeconomic variable was associated with HL level in our study, aligning with different authors [28,29]. In clinical practice, it would be interesting to assess the HL level of each expectant mother to provide tailored information. The standard information we offer to women should be adapted to their level, potentially clarifying vital information to prevent early breastfeeding abandonment [30]. Therefore, including an HL assessment as a healthcare policy could reduce the attrition rate if confirmed by other authors in diverse samples with heterogeneous characteristics [31]. Alternatively, each woman’s level of breastfeeding literacy could be assessed on an individualized and personalized basis through specific instruments [32]. Future studies should assess this aspect in more depth. 

Another facilitating factor for early BF abandonment that was found in our study was immobilization during labour. At first glance, this relationship was not explored in previous studies. We know that mobilization is positively associated with spontaneous vaginal births, as it can help to facilitate the birthing process; relatedly, immobility is linked to an increase in childbirth interventions, and it is related to worse pain management [33,34]. Therefore, birth interventions and difficulty in pain management may increase the perception of lack of self-control, which may increase stress and decrease self-efficacy and satisfaction after childbirth [35], which could negatively affect the mother’s ability or willingness to continue breastfeeding [36]. Regarding other intrapartum variables, we are also aware of aspects that can be directly related to breastfeeding. In particular, it is known that maternal water restriction during labour can be a problem. As stated in the context of the current popularization, no one would think of running a long-distance race without drinking water, but we still apply it to women during labour. It is necessary to add that we are aware and concerned that there are still, in Spain, some intrapartum manoeuvres, such as the Kristeller manoeuvre, that are not being correctly registered [37]. Therefore, other variables may not have been recorded and could have been related to the results obtained. This relationship should be explored in future studies to test this hypothesis.

Finally, labour induction is positively associated with early weaning of BF. Similar to mobilization, induced labour is linked to a higher number of dystocic births and specifically increases the rate of Caesarean sections compared with spontaneous labour [38]. Labour induction often involves the administration of medications and medical procedures to initiate or expedite the birthing process [39]. This may lead to a potentially more intense childbirth experience compared with spontaneous labour. The additional stress and more intense experience could influence the mother’s willingness and ability to initiate and maintain breastfeeding. Previous studies suggested that labour induction can negatively affect the emotional well-being of women in the postpartum period [40,41], which is a factor related to the BF duration in the literature [42,43]. Caesarean sections, especially those performed emergently, may be associated with initial difficulties in breastfeeding initiation due to the need for surgical recovery and other potential factors [44]. Thus, the relationship between labour induction and early BF abandonment may result from a combination of factors related to the birthing experience, potential complications, and the influence on natural hormonal processes that support BF.

This study had several limitations. First, the sample selection was not based on probabilistic sampling and was relatively small, and thus, the results may not represent the general population due to the sample size and selection method. However, sample representativeness was achieved as it exceeded the estimated sample size, and despite the non-probabilistic selection, this fact added robustness to the results. While it is true that our research reflected local practices in Spain, we recognize the importance of emphasizing the novelty and unique contributions our study brings to the existing literature in the field. Our study stands out for its comprehensive exploration of the intricate relationship between HL, obstetric practices, and the duration of BF. The prospective and multicentric nature allowed for a broader perspective, capturing diverse experiences and practices within Spanish regions. 

Second, the data collection method through electronic surveys implied a limitation inherent to the validity of self-reported responses, as these may be subject to subjective interpretation and participant memory bias. Additionally, the possibility of response bias should be considered, where participants may selectively respond or provide socially desirable answers.

Finally, while our study provides insights into breastfeeding practices, it is essential to acknowledge the potential impact of the COVID-19 pandemic. The pandemic has disrupted healthcare systems and society, therefore affecting maternal well-being. These factors may indirectly influence breastfeeding behaviours [45]. However, due to the nature of our data collection, we could not assess the pandemic’s effect on breastfeeding initiation, duration, or exclusivity. Future research should consider prospective designs and explore how pandemic-related stress, isolation, and healthcare access may shape maternal decisions regarding breastfeeding.

## 5. Conclusions

Our findings underscore the importance of considering obstetric and maternal health literacy factors when addressing breastfeeding duration. The research highlights the crucial role of health literacy; spontaneous rupture of membranes; and supportive labour practices, such as mobilization during dilation, in promoting and sustaining breastfeeding. Given the rates were below the WHO recommendations, the need for personalized health literacy assessments and targeted strategies to bridge the gap between current practices and global health guidelines is evident. These findings emphasize the complexity of factors influencing breastfeeding and advocate for specific interventions to enhance maternal and child health outcomes. 

Based on the findings from our study, health stakeholders and policymakers should comprehensively grasp the intricate nature of maternity care. Routine care taken during childbirth can have repercussions beyond the immediate birth; health strategies should be implemented to achieve overall maternal well-being. Healthcare decisions should focus on immediate health outcomes and consider the broader impact on maternity care.

## Figures and Tables

**Table 1:** Sociodemographic and obstetric–neonatal characteristics of the sample (N = 275).

		Early BF Abandonment in the Previous 6 Months				
		No *n* = 117 (42.5%)		Yes *n* = 158 (57.5%)		
		*n*	%	*n*	%	*p*-Value *
Country of origin	Spain	108	92.3	140	88.6	0.308
	Foreign	9	7.7	18	11.4	
Education level	Primary to secondary school	54	46.2	73	46.2	0.994
	University	63	53.8	85	53.8	
Civil status	Others	37	31.6	55	34.8	0.994
	Married	80	68.4	103	65.2	
Employment status	Unemployed or student	38	32.5	59	37.3	0.994
	Employee or professional	79	67.5	99	62.7	
Economic status	<EUR 1000/month	50	42.7	74	46.8	0.499
	>EUR 1000/month	67	57.3	84	53.2	
Financial stability level	Bad or medium	51	43.6	83	52.5	0.142
	Good or very good	66	56.4	75	47.5	
Desired type of breastfeeding	Exclusive	110	94	138	87.3	0.177
	Mixed	5	4.3	13	8.2	
	Not desired yet	2	1.7	7	4.4	
Previous breastfeeding information	No information	4	3.4	10	6.3	0.093
	Family or friend	21	17.9	47	29.7	
	Healthcare professional	34	29.1	36	22.8	
	Books	10	8.5	5	3.2	
	Birth preparation	19	16.2	18	11.4	
	Breastfeeding group	1	0.9	1	0.6	
	Digital tools	28	23.9	41	25.9	
Health literacy level by HLS-EU-16Q	Inadequate	28	23.9	56	35.4	0.040
	Adequate	89	76.1	102	64.6	
Parity	Nulliparous	81	69.2	121	76.6	0.172
	Multiparous	36	30.8	37	23.4	
Pregnancy risk	Low risk	92	78.6	118	74.7	0.649
	Gestational diabetes	9	7.7	16	10.1	
	Hypothyroidism	4	3.4	5	3.2	
	Preeclampsia/hypertension	2	1.7	2	1.3	
	Infertility	1	0.9	1	0.6	
	Premature birth	0	0	4	2.5	
	Other gestational diseases	8	6.8	8	5.1	
	Chronic condition with medication	1	0.9	4	2.5	
Onset of labour	Spontaneous or elective C-section	95	81.2	100	63.3	0.014
	Induction	22	18.8	58	36.7	
Type of rupture of membranes	Spontaneous	83	70.9	89	56.3	0.013
	Artificial	34	29.1	69	43.7	
Streptococcus Agalactie B	Negative	96	82.1	129	81.6	0.931
	Positive	21	17.9	29	18.4	
Intrapartum use of antibiotic	No	98	83.8	126	79.7	0.397
	Yes	19	16.2	32	20.3	
Type of analgesia	Inhalator	0	0	0	0	0.420
	Local	3	2.6	8	5.1	
	Epidural	92	78.6	123	77.8	
	Without analgesia	9	7.7	16	10.1	
	Spinal	13	11.1	11	7	
Kristeller manoeuvre	No	103	88	145	91.8	0.303
	Yes	14	12	13	8.2	
Drinking allowed during labour	No	28	23.9	57	36.1	0.031
	Yes	89	76.1	101	63.9	
Labour accompaniment	No	8	6.8	6	3.8	0.257
	Yes	109	93.2	152	96.2	
Mobilization allowed during labour	No	27	23.1	57	36.3	0.019
	Yes	90	76.9	100	63.7	
Positioning in birth (*n =* 218)	Vertical	8	8.4	21	17.1	0.087
	Lithotomy	61	64.2	81	65.9	
	Lateral decubitus	26	27.4	21	17.1	
Type of birth	Spontaneous vaginal	66	56.4	90	57	0.443
	Instrumental vaginal	30	25.6	32	20.3	
	C-section	21	17.9	36	22.8	
Type of instrumental birth	Vacuum	26	86.7	24	75	0.472
	Spatulas	2	6.7	5	15.6	
	Forceps	2	6.7	3	9.4	
Episiotomy	No	68	70.8	81	66.4	0.484
	Yes	28	29.2	41	33.6	
Perineum injury	Intact	19	27.9	27	32.5	0.846
	Grade I	28	41.2	29	34.9	
	Grade II	20	29.4	25	30.1	
	Grade III	1	1.5	2	2.4	
Sex of newborn	Female	63	53.8	75	47.5	0.296
	Male	54	46.2	83	52.5	
Early skin-to-skin contact	No	2	1.7	7	4.4	0.288
	Yes	108	92.3	137	86.7	
	Companion	7	6	14	8.9	
Breastfeeding initiation	<2 h	90	76.9	109	69	0.146
	>2 h	27	23.1	49	31	

* Chi-square test; significant *p*-values < 0.05. C-section: Caesarean section; HLS-EU-16Q: health literacy survey European Union short questionnaire in Spanish.

**Table 2:** Kaplan–Meier survival analysis for the duration of breastfeeding (N = 275).

	Mean				Median		Log Rank (Mantel–Cox)		
	Estimation	SE	95% Confidence Interval		Estimation	SE	Chi-Square	*df*	*p*-Value
			Lower Limit	Upper Limit					
HL level									
Inadequate	89.17	8.18	73.13	105.2	80	28.41	6.615	1	0.01
Adequate	116.44	5.07	106.5	126.38	132				
Global	108.11	4.38	99.52	116.7	123	7.77			
Onset of labour									
Spontaneous or elective C-section	112.74	5.37	102.22	123.26	145		8.25	1	0.004
Induction	96.83	7.33	82.45	111.2	110	11.72			
Global	108.11	4.38	99.52	116.7	123	7.77			
Type of rupture of membranes									
Spontaneous	111.27	5.75	100	122.53	140		3.979	1	0.046
Artificial	102.83	6.67	89.77	115.9	114	8.46			
Global	108.11	4.38	99.52	116.7	123	7.77			
Drinking allowed during labour									
No	92.4	8.01	76.69	108.11	99	20.49	5.513	1	0.019
Yes	115.14	5.15	105.04	125.24	130				
Global	108.11	4.38	99.52	116.7	123	7.77			
Mobilization allowed during labour									
No	91.42	8.06	75.63	107.2	98	20.62	6.833	1	0.009
Yes	115.43	5.16	105.31	125.54	132				
Global	108.07	4.4	99.44	116.69	123	8.01			

Significant *p*-values < 0.05.

**Table 3:** Cox regression analysis for predicting early breastfeeding abandonment.

	B	SD	Wald	*df*	*p*-Value	Exp(B)
HL level	−0.384	0.168	5.257	1	0.022	0.681
Mobilization allowed during labour	−0.392	0.167	5.537	1	0.019	0.676
Type of onset of labour	0.431	0.167	6.640	1	0.010	1.538

SD: standard deviation; df: degrees of freedom; Exp(B): odds ratio; significant *p*-values < 0.05. The statistical significance and Exp(B) values provide insights into the magnitude and direction of the associations, supporting the relevance of these factors in understanding early BF abandonment.

## Data Availability

Data are available upon reasonable request. All necessary data are supplied and available in the manuscript; however, the corresponding author will provide the dataset upon request.

## Supplementary Materials

The following supporting information can be downloaded from https://www.mdpi.com/article/10.3390/nu16050690/s1—Table S1: HLS-EU-Q16 questionnaire, Spanish version.
