# Supplementary material for: Factors Influencing Duration of Breastfeeding: Insights from a Prospective Study of Maternal Health Literacy and Obstetric Practices
Source: Nutrients. 2024 Feb 28;16(5):690. doi: 10.3390/nu16050690 (PMC10933905; doi:10.3390/nu16050690)
Supplement: Supplementary file 1 [file nutrients-16-00690-s001.zip › nutrients-2888762-supplementary.pdf]

The HLS-EU-Q16 questionnaire consists of 16 questions that classify the perceived difficulty level of the respondent for each task or situation as very easy, easy, difficult, very difficult or do not know/do not answer.

Supplementary Table S1. HLS-EU-Q16 questionnaire, Spanish version.

1. Encontrar información sobre los tratamientos asociados a las enfermedades que son de su interés
2. Averiguar dónde conseguir ayuda profesional cuando se encuentra enfermo (p. ej., médico, farmacéutico o psicólogo)
3. Entender lo que le dice el médico
4. Entender las instrucciones del médico o farmacéutico sobre cómo tomar las medicinas recetadas
5. Valorar cuándo puede necesitar una segunda opinión de otro médico
6. Utilizar la información proporcionada por el médico para tomar decisiones sobre su enfermedad
7. Seguir las instrucciones de su médico o farmacéutico
8. Encontrar información sobre la manera de abordar problemas de salud mental, como el estrés o la depresión
9. Comprender las advertencias sanitarias relacionadas con hábitos como fumar, hacer poco ejercicio físico o beber alcohol en exceso
10. Comprender por qué necesita hacerse pruebas de detección precoz de enfermedades o chequeos médicos (p. ej., mamografía, prueba de azúcar en sangre y presión arterial)
11. Valorar la fiabilidad de la información sobre riesgos para la salud que aparece en los medios de comunicación (p. ej., televisión, Internet u otros medios de información)
12. Decidir cómo protegerse de las enfermedades gracias a la información que proporcionan los medios de comunicación (p. ej., periódicos, folletos, Internet u otros medios de información)
13. Encontrar actividades que sean buenas para su bienestar mental (p. ej., meditación, ejercicio, paseos, pilates, etc.)
14. Comprender los consejos sobre salud que dan la familia y los amigos
15. Comprender la información proporcionada por los medios de comunicación sobre cómo mejorar su salud (p. ej., Internet, periódicos, revistas)
16. Valorar cuáles de sus hábitos diarios afectan a su salud (p. ej., costumbres relacionadas con el consumo de alcohol, hábitos alimenticios, ejercicio, etc.)

Each response is dichotomous, assigning the following scores: very difficult and difficult = 0, easy and very easy = 1. The score for each participant is obtained by summing the scores of the 16 items, considering "inadequate level" (score between 0 and 12) and "adequate level" (score between 13 and 16).
